# Investigating the Parental and Media Influence on Gender Stereotypes and Young Student’s Career Choices in Pakistan

**DOI:** 10.3389/fpsyg.2022.890680

**Published:** 2022-06-28

**Authors:** Mehdi Hassan, Yingzi Luo, Jianxiu Gu, Iqra Mushtaque, Muhammad Rizwan

**Affiliations:** ^1^College of Public Administration, Nanjing Agricultural University, Nanjing, China; ^2^Department of Psychology, Bahauddin Zakariya University, Multan, Pakistan; ^3^Department of Sociology, Nanjing Normal Univeristy, Nanjing, China

**Keywords:** parental influence, media richness, gender stereotype, career choice, secondary school students

## Abstract

The study aimed to examine the impact of parental influence and media richness on gender stereotypes and career decisions among students at the secondary level in Pakistan. The sample size was 200 students, selected through a simple random sampling technique from government and private schools. Four questionnaires were used to gather data. The data was analyzed quantitatively using the Statistical Package for the Social Sciences (SPSS). Regression analyses were used to investigate the impact of parental influence (β = 0.50) on gender stereotypes and media richness influence (β = 0.26) on gender stereotype beliefs. Furthermore, parental, media, and gender stereotype behavior all have a significant impact on students’ career choices (*R*^2^ = 0.694). On the scale of the parental influence and media richness, no significant gender differences were found. It is concluded that parental influence has a greater effect on students’ gender stereotyping behavior and career choices.

## Introduction

In the twenty-first century, more students have demonstrated an interest science, technology, engineering, and mathematics (STEM). Female students outnumber male students in various STEM fields, including biology, medicine, Physics, and chemistry (Wieman, 2013). Many gender differences in subject and job choice, according to study, are related to social prejudices rather than intelligence differences between men and women (Cheryan et al., 2017). Girls and boys undergo different socialization processes, (Bian et al., 2017) such as the gender stereotypes they face in their contexts, and television programs may constitute gender stereotyping endorsement among children’s (Wille et al., 2018). According to stereotype threat research, the stereotype that females perform worse in STEM than males can harm girls’ STEM performance and motivation, but the stereotype that males perform better in STEM can help boys’ STEM performance (Spencer et al., 2016). According to HESA’s most recent data (2017–2018), 35% of women enrolled in STEM subjects in higher education in the United Kingdom, yet they continue to be underrepresented in comparison to men (Stem Statistic, 2021). Women enrolled in only 17% of Engineering and Technology programs and 19% of Computer Science programs in 2016–17, but 75% of Medicine, Dentistry, and Veterinary Science subjects (Scottish Funding Council, 2018). In Scotland, the gender disparity in essential STEM jobs held by women is narrowing slightly, growing from 39% in 2010 to 42% in 2016. As with other subjects in education, the amount varies significantly amongst STEM fields. Numerous professions continue to be dominated by men; only 19% of engineers in Scotland and only 3% of licensed civil engineers are female (Close the Gap, 2018). Similarly numerous economic and social decisions are influenced by our attitudes toward ourselves and others. Von Hippel et al., 2011 men and women’s abilities are frequently found to be distorted by such stereotypes. Women are less confident in their mathematical and scientific ability than men, which contribute to economically important disparities in financial decision-making, academic accomplishment, and career choices (Buser et al., 2014). A significant change is school children were found after the gender stereotype related knowledge and endorsement in United Kingdom. Gender stereotyping is ubiquitous, with male and female categorization emphasized to children in particular as a dominant societal framework, despite growing public support for equal opportunities for boys and girls (Ellemers, 2018).

Career choice is a crucial decision that will impact the remainder of an individual’s life. Career exploration is the process of examining and weighing various work opportunities. According to El-Hassan and Ghalayini (2019) says that investigating job opportunities before to making a commitment boosts future professional achievement and contentment. Parental attachment is related to job exploration. They discovered a link between parental attachment and vocational exploration. Similarly, Lee et al. (2012) observed a positive relationship between parental attachment and professional maturity, which is defined as an adolescent’s readiness to make career decisions. Professional exploration is an important stage in the career development process.

Kirdök and Korkmaz (2018) parenting teenagers is generally portrayed as a difficult task. Before becoming adults, teenagers go through a number of developmental changes, including biological, cognitive, emotional, and social changes. Effective teen parenting necessitates a thorough understanding of these natural developmental shifts. Knowing that their parenting style is a basis for many favorable adolescent developmental outcomes might be beneficial to parents. Understanding the various parenting styles and how they affect the parent-teen relationship can assist parents and their adolescent children in navigating adolescence (El-Hassan and Ghalayini, 2019).

The pandemic of Coronavirus Disease in 2019 (COVID-19) has put educational systems and students under unprecedented strain (Mushtaque et al., 2021). Furthermore, the epidemic had an impact on students’ plans for their future careers. One-fifth students reported that COVID-19 cause inclination to choose to be a doctor, on the other side COVID-19 pandemic improved students’ willingness and determination to specialize in respiratory medicine and infectious diseases. Females were more influenced by the epidemic in terms of key decisions, such as being frightened of change and taking chances. Following the college admissions exam, the majors chosen by medical students are determined by their parents, social orientation, and professional income. Female college students’ customary role expectations run counter to modern egalitarian culture, which places a premium on self-realization. They are easily able to transfer majors when confronted with enormous challenges such as the COVID-19 pandemic (Deng et al., 2021; Gong et al., 2021). Throughout the first lockdown, fathers assumed the major caretaker role in many United Kingdom households, inverting conventional gender roles. However, in certain houses, traditional gender roles were more established, with mothers being obliged to stay at home during the second lockdown while men were permitted to work (Hupkau and Petrongolo, 2020).

Prior study indicates a causal relationship between gender stereotyping behavior and occupational choices. Knowledge endorsement has a significant favorable effect on the modification of career stereotypes. However, new phenomena such as COVID-19 induced stress, necessitating a thorough understanding of the mechanisms activated by stereotypes under stressful settings. Stereotypes play a unique role in influencing our judgment of good and wrong. It establishes gender roles and has a significant impact on socialization. In the current study we examined the Pakistani population to examine the variable. The purpose of the study was to explore the influence of parental and social media richness on adolescent gender stereotype beliefs and their career choices in Pakistan. Specifically, the study sought to know about the available knowledge of career information among secondary school students and sought to identify different sources of career information for students. The impact of parental influence and media richness on students’ gender stereotype perception was also examined. The choice of career among students was investigated with parental influence, media richness, and their perception of gender stereotypes.

## Theoretical Framework

### Social Cognitive Career Theory

Social cognitive career theory (Lent et al., 1994) has been found to be a valuable framework for examining job choice processes in previous studies. As demonstrated by Cunningham et al. (2005) and Alexander et al. (2010), SCCT provides a social and heuristic framework for assessing academic and professional decisions. The gender gap in STEM has been studied extensively (Park et al., 2018). A description of Bandura and Cervone (1986) broad social cognition theory’s three key tenets: self-efficacy, expectations, and goal representations (Bandura, 2002; Lent and Brown, 2013).

### Social Learning Theory

American scholars created the social learning theory (SLT). This idea says four things impact career choices. These are genetics, environment, learning, and task-approach skills. Gender, ethnicity, color, IQ, particular ability, and so forth are all examples of genetic endowment. Environmental influences include family, teachers and resources, technology, training possibilities, occupational factors, labor market, and so on. The factors influencing a person’s professional choices are largely out of their control. Instrumental and associative learning experiences can influence career choices. Task approach skills include learning skills, goal setting, and getting occupational knowledge. According to the SLT, a person’s profession choice is influenced by self-reported interests, abilities, and capabilities, as well as assumptions about the workplace (Barletta, 1999; Krumboltz and Worthington, 1999; Di Palma and Reid, 2021).

**FIGURE 1:**
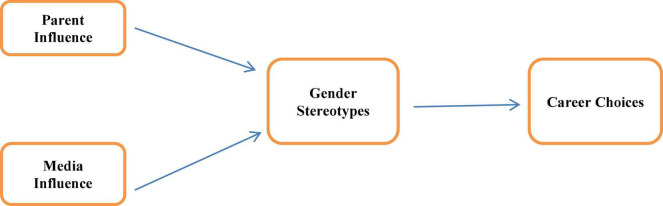
Conceptual framework of the study.

The purpose of the study was to investigate the influence of parental and social media richness on adolescent gender stereotype beliefs and their career choice in Pakistan. Specifically, the study sought to know about the available knowledge of career information among secondary school students, and seek to identify different sources of career information for students. The impact of parental influence and media richness is indicative of students’ gender stereotype perception; also the choice of career among students was investigated with parental influence, media richness, and their perception of gender stereotype. Gender differences concerning parental influence, media richness, and gender stereotype perception were also a purpose of the study. The main purpose of the research was to study the parental and social media inspiration on gender stereotype perception and career choice among students.

### Hypothesis of the Study

H1. Parental influence is likely to predict gender stereotyping behavior among students.

H2. Media influence is likely to predict gender stereotyping behavior among students.

H3: parental influence, media influence, and gender stereotype all influence students’ career choices.

H4: There are likely to be gender differences on the scale of parental influence, media influence, gender stereotypes and career decisions.

### Methodology

This study was carried out to investigate the effect of parental influence and media richness on gender stereotyping and career choice among secondary school students in Pakistan. The quantitative approach was used to obtain data from students. A quantitative approach is the best choice to gather objective information and helps to generalize the outcomes of the study. The current study is based on a correlational design. The simple random sampling technique was used as Random sampling guarantees that the results obtained from the sample are close to results obtained if the full population was surveyed (Shadish, 2002). The simplest random sample gives each unit in the population an equal probability of being chosen. Perhaps the most important benefit to selecting random samples is that it enables the researcher to rely upon assumptions of statistical theory to conclude from what is observed. The sample size consists of 200 students, ages ranged 16–21 years who were enrolled in different private and government schools. The level of confidence is 95% for this study. A simple random sampling technique was used to approach students. Statistical Package for Social Science (SPSS) software was used to analyze data. Descriptive and inferential statistics were used to proceed with the results.

### Instruments

The instrument consists of six scales to gather data.

**(1) Parental Influence scale** was developed by Wong and Liu (2010). Six-item scale use to measure the degree of parental influence. Higher scores indicate a higher level of parental influence. A 5-point Likert scale ranging from (1) strongly disagree to (5) strongly agree was used to record the response rate of students.
**(2) Media Richness scale** was developed by kohring and Lin in 2010. Response rates were on a 5-point Likert scale, ranging from 1 = strongly disagree to 5 = strongly agree.
**(3) Traditional Gender Stereotypes scale** was developed to measure stereotyping gender roles as associating women with warmth, and men with competence (Runge et al., 1981). Responses are on a 5-point Likert scale, anchored 1 = “applies more to men,” 5 = “applies more to women,” (Cronbach’s α = 0.83).
**(4) Career-related Stereotype scale** was developed by Fuegen and Endicott in 2010. It is used to measure stereotype perception related to career choice.

## Results

The findings were presented by the use of descriptive and inferential statistics.

### Identification of Various Professions/Career

The objective is to know about the knowledge students have about the different types of professions at the secondary school level. The students were asked to circle or tick the profession they want to adopt in later years. A list of seven professions was provided and students have to mark one profession that they want to choose in their life for themselves.

The frequency table shows the greatest percentage goes with doctor/medical career [29.0% preference, followed by engineering, (21.5%), pilot (12.5%), army 99.0%], accounting (7.0%), and then law (5.5%), respectively. The students were aware of these professions and plan to pursue a certain career of their choice.

### Source of Career Choices

In the follow-up question, students were asked to indicate their sources of career information. A list of seven sources was provided and students were asked to tick the source of their preferable career choice.

The frequency table shows the greatest percentage goes with electronic media (27.0%), then parents (26.0%), friends (17.0%), print media (11.5%), teachers (8.5%), role models (5.5%), and Twitter (4.5%). The findings show the major source of career information are electronic media and parents, as some students do not have the choice of the internet and social media hence they rely on their parents, teacher, and friends in selecting their careers. Students are active on Facebook and Instagram but not very active on Twitter therefore their career choice is mostly dependent on electronic media.

### Impact of Parental Influence and Media Richness on Gender Stereotypes

Table 3 shows the impact of parental influence and media richness on gender stereotypes among students. A multiple linear regression was calculated to predict gender stereotypes based on parental influence and media richness. A significant regression was found with *r*^2^ of parental influence (*r*^2^ = 0.31) and media richness (*r*^2^ = 0.30). Young students predicted that gender stereotypes increased by 0.67 due to parental influence. While young students predicted that gender stereotypes affected 0.12 due to media richness, which is less from parental influence. As a result, the findings confirm that young children learn patterns from their parents. These results also support the theory of social learning; children learn and perform the actions that they see their parents do.

**TABLE 1:** Choice of career.

Professions	Frequency	Percent
Army	18	9.0
Engineering	43	21.5
Doctor	58	29.0
Teacher	31	15.5
Pilot	25	12.5
Law	11	5.5
Accounting	14	7.0
Total	200	100.0

**TABLE 2:** Source of career information (*N* = 200).

Professions	Frequency	Percent
Print media	23	11.5
Fiends	34	17.0
Electronic media	54	27.0
Parents	52	26.0
Teachers	17	8.5
Twitter	9	4.5
Role model	11	5.5
Total	200	100.0

**TABLE 3:** Multiple regression of parental influence and media richness on gender stereotype among students (*N* = 200).

Predictor	*B*	Std. error	Beta	*t*	*p*-value
(Constant)	10.149	2.228		4.555	0.000
Parental influence	0.676	0.079	0.506	8.570	0.000
Media richness	0.120	0.026	0.269	4.560	0.000

*R^2^ = 0.314, Adjusted R^2^ = 0.308.*

### Impact of Parental Influence, Media Richness, and Gender Stereotypes on Career Choice

Table 4 shows the impact of parental influence and media richness on career choice among students. A multiple linear regression was calculated to predict career choices based on parental influence, media richness, and gender stereotype. Young students predicted that career choice decisions were due to parental influence (1.36), media riches (-0.32) and gender stereotypes (1.18). The results revealed that the career decisions of the students depended on their parents’ choices and the students’ own perceptions regarding gender stereotypes. The results show that the media did not have a significant contribution to shaping the students’ decisions regarding their career choices in developing countries like Pakistan.

**TABLE 4:** Multiple regression analysis of parental influence, media richness, and gender stereotypes on career choice among students (*N* = 200).

Predictor	*B*	Std. error	Beta	*t*	*p*-value
(Constant)	5.764	3.692		1.561	0.120
Parental influence	1.368	0.146	0.435	9.390	0.000
Media richness	–0.325	0.044	–0.310	–7.449	0.000
Gender stereotype	1.186	0.112	0.504	10.563	0.000

*R^2^ = 0.694, Adjusted R^2^ = 0.689.*

## Gender Differences in Parental Influence, Media Richness, Gender Stereotype, and Career Choice

Table 5 describes the mean differences in parental influence, media richness, gender stereotypes, and career choice among students concerning their gender (male and female). The comparisons with respect to gender were found to be insignificant (*P* > 0.05) on the parental influence and media richness scales, while the results were found to be significant with respect to gender stereotypes (*t* = -2.98, *p* = 0.003) and career decisions (*t* = -3.79, *p* = 0.000) among students at the secondary level. Male has low mean score on the scale of gender stereotype while female has high score on the scale of gender stereotyping behavior. It was found that female students had more gender stereotypes and were surer of them when making career decisions than male students.

**TABLE 5:** Mean, standard deviation, *t*-value, and scores of parental influence, media richness, gender stereotype, and career choice among students (*N* = 200).

Variables	Gender	*N*	*M*	*SD*	df	*t*-test	*p*-value
Parental influence	Male	135	19.5556	3.65829	198	–1.802	0.074
	Female	65	20.4462	3.07221			
Media richness	Male	135	56.8370	10.11738	198	1.164	0.247
	Female	65	54.9385	11.12272			
Gender stereotype	Male	135	29.6444	4.59309	198	–2.986	0.003
	Female	65	31.7077	4.54396			
Career decision	Male	135	48.6074	11.32893	198	–3.790	0.000
	Female	65	54.6923	9.00507			

## Discussion

The findings of students’ career choices show secondary school students’ preferences for various careers. The majority of students want to be doctors (Gaurav and Sheikh, 2020), with engineering coming in second (Virtič and Šorgo, 2022). Although these two occupations have been famous for decades and are often regarded as the most respected and well-paid, there is no doubt that they are excellent options (Eagly and Wood, 1999). However, there are several developing professions with high prestige and salaries. Even though not every student has the ability to pursue these careers, they are the most popular among students. The teaching profession was not a high-career choice, which is alarming because good and knowledgeable teachers are needed. The most important sources of career information are electronic media and parents (Ulrich et al., 2018). Students are unaware of other career choices available at this time and they have limited sources of career information. There is no proper career counseling facility available for children. While at this stage of life, they need proper occupational counseling that helps to choose the right career/professional according to their abilities and aptitude. Results indicate that some students had no access to journals, newspapers, or the internet, so they relied on teachers and parents (Owen et al., 2020). Social media promotes every type of career choice without providing further details about the input during the career-related degrees (Makela and Hoff, 2019). Furthermore, parents have limited knowledge about various professions and attempt to pursue the child solely by observing a few role models in specific careers. Parental influence (Eisend, 2019) and media richness predict gender stereotypes at a significant level. Students mostly interact with their parents and spend time on social media. The stereotype perception about gender is not innate; it’s learned over time. Parental influence and media richness (Cao et al., 2021) are two factors that are responsible for gender stereotype perception among students. There are gender stereotypes in daily life, in career choices, in social strata, and in every sphere of life. For example, males do not want to be nurses in society, and parents and peers do not think it’s right for males to be nurses. Similarly, females are perceived to have low intelligence in mathematics and science subjects. These gender stereotypical perceptions limit the capabilities of the younger generation. The impact of parental influence, media richness, and gender stereotypes on career choice is massive. These variables predict career choice among students. The students are aware of their assigned gender roles and of their career preferences. It means students know to differentiate their gender roles and pursue some sort of career. Therefore, traditional career choice is predicted by parental influence (Venant et al., 2021), media richness (Kumar and Nanda, 2019), and gender stereotype perception. Gender stereotypes and career decisions were found to be more prevalent in female students than in male students. There is no difference in parenting styles between men and women. Pakistan has a collective culture, and parents try to impose their will on their children. They try to teach their children directly or indirectly at this stage of life, so their influence on children is similar regardless of the child’s gender. In the case of media richness, the availability of content is similar for males and females. Gender stereotypes and career choices reveal significant gender differences. Males have masculine career preferences, whereas females have stereotypically feminine career preferences. Females perceive gender stereotypes more strongly than males. Similarly, at the secondary school level in Pakistan, females have higher career choice preferences than males.

## Conclusion

The persistent impact of parental influence, media richness, and gender stereotypes was found on students’ career choices at the secondary level. Students know about different careers and most students want to pursue medical, engineering, and pilot careers as their future career choices. The top three sources of information according to children are electronic media, parents, and friends. Regression analysis shows a significant impact of parental influence and social media influence on gender stereotypes and, in turn, career choice. The study found no gender difference in parental influence. It might be the reason that parents try to treat their children equally and try to influence their children regardless of their gender. Similarly, media richness shows non-significant gender differences, which might be because the media richness is for all individuals regardless of their gender. On the other hand, gender differences in gender stereotype perception and career choice were found to be significant. Based on the findings of the study, parental influence, media richness, and gender stereotypes predict career choices among secondary school students in Pakistan.

### Practical Implications

The current study, carried through cross-sectional design, explored the influence of parenting and media richness. This article indicates the impact of parental influence and media richness on gender stereotypes and career choices. According to empirical studies, gender stereotypes influence how people pay attention to, perceive, and recall information about themselves and others. By considering the cognitive and motivational functions of gender stereotypes, we can better comprehend the influence of gender stereotypes on implicit beliefs and communication about men and women. Knowledge of the literature on this topic might help people make more informed decisions in settings where gender stereotypes are likely to be present. These findings add to our knowledge of the roles of parental influence and media richness in determining adolescent profession choices and show that gender stereotypes have a cumulative effect on career choices.

### Limitations of the Study

The current study is informative yet contains many limitations. The cross-sectional research design for this study is limited in its generalization power. Another limitation is the sample size, as researchers have constrained resources. Additional important information on parents and families that may impact parental school experiences, such as parents’ gender, geographic location, or school type (e.g., urban, rural, or suburban), was not included in the available data. One limitation is that the credibility of information on social media has not been considered.

### Direction for Future Studies

A longitudinal study with a larger sample size is needed to investigate the impact of parents on gender stereotypes and career choices. As a result, it is suggested that the study be repeated with different parenting styles. Parents should practice not passing on gender stereotypes to their children. Similarly, pupils must be taught that they may succeed in various vocations and in society regardless of their gender. Parents will be expected to take steps to teach their children not to distinguish between biological and gender roles. Secondly, parents and social media are identified as the primary sources of professional choice due to the lack of career guidance at the school level. It is suggested that career counseling sessions be introduced to educate not only students but also their parents from various sources. Finally, rigorous study with a holistic approach to analyze numerous aspects of gender stereotyping behavior and profession choice will be advised.

## Data Availability Statement

The original contributions presented in this study are included in the article/supplementary material, further inquiries can be directed to the corresponding author.

## Ethics Statement

Ethical review and approval was not required for the study on human participants in accordance with the local legislation and institutional requirements. Written informed consent to participate in this study was provided by the participants or their legal guardian/next of kin.

## Author Contributions

All authors listed have made a substantial, direct, and intellectual contribution to the work, and approved it for publication.

## Conflict of Interest

The authors declare that the research was conducted in the absence of any commercial or financial relationships that could be construed as a potential conflict of interest.

## Publisher’s Note

All claims expressed in this article are solely those of the authors and do not necessarily represent those of their affiliated organizations, or those of the publisher, the editors and the reviewers. Any product that may be evaluated in this article, or claim that may be made by its manufacturer, is not guaranteed or endorsed by the publisher.
